# Behavioral Phenotype of *Fmr1* Knock-Out Mice during Active Phase in an Altered Light/Dark Cycle^1^,^2^,^3^

**DOI:** 10.1523/ENEURO.0035-16.2016

**Published:** 2016-05-03

**Authors:** R. Michelle Saré, Merlin Levine, Carolyn Beebe Smith

**Affiliations:** Section on Neuroadaptation and Protein Metabolism, Department of Health and Human Services, National Institute of Mental Health, National Institutes of Health, Bethesda, Maryland 20892

**Keywords:** activity, anxiety, circadian phase, fragile X, learning and memory, social behavior

## Abstract

Fragile X syndrome (FXS) is the most commonly inherited form of intellectual disability and is a disorder that is also highly associated with autism. FXS occurs as a result of an expanded CGG repeat sequence leading to transcriptional silencing. In an animal model of FXS in which *Fmr1* is knocked out (*Fmr1* KO), many physical, physiological, and behavioral characteristics of the human disease are recapitulated. Prior characterization of the mouse model was conducted during the day, the inactive phase of the circadian cycle. Circadian rhythms are an important contributor to behavior and may play a role in the study of disease phenotype. Moreover, changes in the parameters of circadian rhythm are known to occur in FXS animal models. We conducted an investigation of key behavioral phenotypes in *Fmr1* KO mice during their active phase. We report that phase did not alter the *Fmr1* KO phenotype in open field activity, anxiety, and learning and memory. There was a slight effect of phase on social behavior as measured by time in chamber, but not by time spent sniffing. Our data strengthen the existing data characterizing the phenotype of *Fmr1* KO mice, indicating that it is independent of circadian phase.

## Significance Statement

This study seeks to characterize the behavioral phenotype of *Fmr1* KO mice during the active circadian phase. Given that for many behaviors the active phase is more physiologically relevant, our study is an important validation of *Fmr1* KO mice as a model for FXS. We find that classical behavioral phenotypes; such as hyperactivity, reduced anxiety, and learning and memory impairments; reported in the *Fmr1* KO mice are not influenced by circadian phase.

## Introduction

Fragile X syndrome (FXS) is the most commonly inherited form of intellectual disability, primarily affecting males with a prevalence of ∼1 in 4000 boys (Turner et al., 1996). Additionally, between 15% and 60% of patients with FXS receive a diagnosis of being on the autism spectrum (Hagerman et al., 1986, 2010; Bailey et al., 1998). Patients with FXS account for about 5-8% of cases of autism (Muhle et al., 2004; Schaefer and Mendelsohn, 2008). Behavioral symptoms present in patients with FXS include the following: intellectual disability, anxiety, hyperactivity, social anxiety, and repetitive behaviors.

FXS is caused by an expanded CGG repeat sequence in the 5'-UTR of the *FMR1* gene, which leads to transcriptional silencing and subsequent loss of the gene product fragile X mental retardation protein (Verheij et al., 1993). This has been modeled in the mouse by deletion of the *Fmr1* gene (*Fmr1* KO). These mice recapitulate many of the clinical features, including physical, physiological, and behavioral, found in FXS patients (Kazdoba et al., 2014).

One important limitation of the phenotyping of FXS mice, particularly with regard to behavior, is that, to our knowledge, all studies have conducted behavior testing during the day, the inactive phase for mice. Given the circadian control of many physiological factors, including body temperature, corticosterone levels, hormones, gene expression, glucose metabolism, immune function, and sleep (Chung et al., 2011; Bass, 2012; Scheiermann et al., 2013), it is reasonable to expect that the circadian phase might also be a strong contributor to behavior. Indeed, the circadian phase has been shown to influence certain behaviors in rodents (including activity, anxiety, and learning; Griebel et al., 1993; Jones and King, 2001; Bertoglio and Carobrez, 2002; Andrade et al., 2003; Hossain et al., 2004; Valentinuzzi et al., 2004), but may not affect others such as social behavior (Hossain et al., 2004; Yang et al., 2008). Circadian rhythm abnormalities are seen in both the *Drosophila* FXS model and *Fmr1* KO mice (Dockendorff et al., 2002; Zhang et al., 2008). We considered the possibility that the circadian cycle might differentially affect *Fmr1* KO mice and confound our understanding of the behavioral phenotype.

In the present study, we sought to determine whether the behavioral abnormalities reported in *Fmr1* KO mice are also evident during the active circadian phase. We housed animals in an altered light/dark environment for at least 1 month prior to testing, and we performed open field, elevated plus maze (EPM), passive avoidance, and social behavior tests during the latter half of the active (dark) phase. We found that, in the active phase, *Fmr1* KO mice have the same phenotype as reported in the inactive phase in open field activity, anxiety, and learning and memory. There was a slight effect of phase on social behavior, but this was reflected only in time in chamber and not in time spent sniffing. These results are an important verification of the mouse model of FXS, showing that they share many features associated with clinical FXS.

## Materials and Methods

### Animals

These studies were conducted on male *Fmr1* KO and control mice (on a C57BL/6J background), which were generated through heterozygous female and control male breeding pairs maintained in-house. The original B6.129P2-Fmr1^tm1Cgr^/J mice were obtained from The Jackson Laboratory (Stock #003025). We have maintained the colony in-house for 6 years, periodically backcrossing back into C57BL/6J mice (Stock #000664, The Jackson Laboratory). Pups were weaned between 21 and 23 d of age. Genotyping of mouse tail DNA was performed by means of PCR amplification. All mice were group housed in a standard housing environment with up to five mice per cage in a climate-controlled central facility. Food (NIH-31 rodent chow, LabDiet) and filtered tap water were available to mice *ad libitum*. From birth to 1 month of age, animals were maintained in a standard 12 h light/dark environment (lights on at 6:00 A.M.). At 1 month of age, and throughout behavioral testing, animals were shifted to a 12 h light/dark environment (lights on at 1:00 P.M.). Sixty mice were studied between the ages of 60 and 90 d. All procedures were performed in accordance with the National Institutes of Health *Guidelines on the Care and Use of* Animals and an animal study protocol approved by the National Institute of Mental Health Animal Care and Use Committee.

### Behavior testing

Behavior testing was performed on mice beginning at 60 d of age. Mice were allowed 1 week between tests. Testing was performed between 8:00 A.M. and 1:00 P.M. during the active phase. The testing order was as follows: open field, social behavior, EPM, and passive avoidance. Open field and social behavior tests were conducted in the dark under red light conditions. Due to the nature of the tests, EPM and passive avoidance tests were performed in lighted conditions. Testing procedures are described below.

### Open field

Open field testing was used to determine levels of general activity, as well as anxiety. Activity was measured for 30 min (in 5 min epochs) by means of photobeam detection (Coulbourn Instruments). The total distance traveled and the ratio of center to total distance traveled were analyzed.

### Social behavior

Mice were tested for social behavior by means of an automated three-chamber apparatus. Briefly, the test was performed in three stages, each lasting 5 min, as follows: (1) Habituation: while the doors were open, mice were placed in the center chamber and allowed to freely explore. (2) Sociability: The test mouse was isolated to the center chamber (Chamber 2), while a gender/age-matched stranger was placed inside a social enclosure (Noldus) in either Chamber 1 or 3. The other chamber contained an empty social enclosure. The doors were opened, and the test mouse was allowed to freely explore. The times spent in each chamber were recorded. (3) Social novelty: immediately following the second phase, test mice were isolated back in the center chamber. A novel gender/age-matched mouse was placed in the previously empty social enclosure. Doors were opened, and the test mouse was allowed to freely explore. Measures were taken as in Stage 2. Video recording of the testing allowed for the subsequent recording of sniffing time [determined by close proximity (<4 cm) from the enclosure with the head directed toward the enclosure]. For the social behavior testing conducted in the dark, video recording was performed by means of a UV-detecting camera and additional UV light (PhantomLite).

Social behavior testing was performed in a second group of animals during the inactive phase. These animals were maintained throughout their life in standard 12 h light/dark environment (lights on at 6:00 A.M.). Testing was performed between 1:00 and 3:00 P.M., also in the light. This group of animals did not receive any other testing.

### Elevated plus maze

Mice were tested for general anxiety by means of the EPM. Mice were placed in the center of the apparatus facing an open arm. The times spent in the open arms, closed arms, and the center, were recorded for 5 min. The mouse was considered to be in a particular arm once the head and forepaws had crossed into an area. Testing for the EPM was conducted in the light so that the animal could perceive differences between open and closed arms. Data are presented as the percentage of time spent in the open arms [open arm time/(open arm time + closed arm time)].

### Passive avoidance

Mice were tested for fear-based learning and memory impairments by means of the passive avoidance system (Coulbourn Instruments). The passive avoidance apparatus was composed of a lighted chamber and a dark chamber, separated by an automated door. The floor of the apparatus was capable of delivering an electric shock to the subject. The test was composed of three sessions over 3 consecutive days (24 h apart). (1) In the habituation phase, the mouse was placed in the lighted chamber with the door to the dark chamber closed. After 30 s, the door opened and the mouse was given 10 min to enter the dark chamber. Once the mouse entered the dark chamber, the door closed and the animal was removed. (2) In the training phase, the mouse was placed in the lighted chamber with the door to the dark chamber closed. After 30 s, the door opened to the dark chamber. Once the mouse entered the dark chamber, the door closed and a 0.3 mA electric shock of 1 s duration was delivered. After 15 s, the mouse was removed from the apparatus and allowed 120 s of rest before repeating the training session. (3) In the testing phase, the mouse was placed in the lighted chamber. After 30 s, the door opened to the dark chamber. The latency to enter the dark chamber was recorded for up to 570 s. Given the necessity of a light chamber, passive avoidance training and testing were conducted in the light.

### Statistical analysis

For passive avoidance and EPM, statistical significance was determined by means of a Student’s *t*-test, comparing control and *Fmr1* KO mice. For the other behavior tests, repeated measures ANOVA was used. For open field, genotype was the between subjects factor and epoch the within subjects factor. For social behavior, genotype and phase were the between subjects factors and chamber the within subjects factor. Effects with *p* ≤ 0.05 were considered to be statistically significant (*), although *p* values >0.05 and ≤0.10 are also reported here and are noted on figures with “∼.” Data are reported as the mean ± SEM.

## Results

### *Fmr1* KO mice are hyperactive in the open field during the active phase

**Figure 1.**
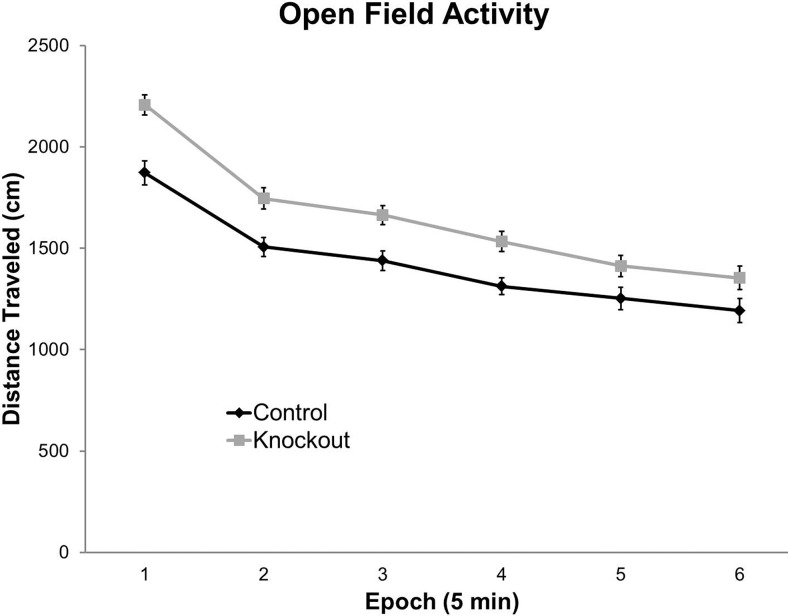
*Fmr1* KO mice display hyperactivity in the open field during the active phase. Both control (n=25) and *Fmr1* KO (n=31) mice display habituation to the novel environment across the 30 min testing period (represented by a significant main effect of epoch). However, at all epochs, *Fmr1* KO mice are hyperactive with respect to controls. This is represented by a statistically significant main effect of genotype. Each point represents the mean +/− SEM for the number of mice indicated in parentheses.

We measured distance traveled in 5 min epochs across 30 min of open field testing (Table 1, Fig. 1). We found a statistically significant main effect of genotype indicating that, overall, *Fmr1* KO mice were hyperactive compared with control mice. We also found a statistically significant effect of epoch, indicating that both control and *Fmr1* KO mice displayed a burst of initial activity in response to the novel environment, and that both groups showed habituation to the environment as testing progressed (Fig. 1).

**Table 1.** Repeated-measures ANOVA results

**Behavior**	**Effect**	***F***_**(df, error)**_ **Value**	***p*** **Value**
Open field			
Total distance moved	Genotype × epoch	*F*_(5,258)_ = 1.417	0.221
	Genotype	*F*_(1,54)_ = 13.943	<0.001^∗^
	Epoch	*F*_(5,258)_ = 123.247	<0.001^∗^
Center/total ratio	Genotype × epoch	*F*_(5,228)_ = 0.594	0.675
	Genotype	*F*_(1,54)_ = 9.620	0.003^∗^
	Epoch	*F*_(5,228)_ = 1.164	0.328
Social behavior			
*Sociability*			
Chamber time	Phase × genotype × chamber	*F*_(1,99)_ = 0.080	0.778
	Genotype × chamber	*F*_(1,99)_ = 0.040	0.842
	Phase × chamber	*F*_(1,99)_ = 0.102	0.750
	Phase × genotype	*F*_(1,99)_ = 0.047	0.829
	Phase	*F*_(1,99)_ = 0.497	0.483
	Genotype	*F*_(1,99)_ = 1.016	0.316
	Chamber	*F*_(1,99)_ = 32.244	<0.001^∗^
Sniffing time	Phase × genotype × chamber	*F*_(1,98)_ = 0.442	0.508
	Genotype × chamber	*F*_(1,98)_ = 0.823	0.367
	Phase × chamber	*F*_(1,98)_ = 0.073	0.787
	Phase × genotype	*F*_(1,98)_ = 0.414	0.522
	Phase	*F*_(1,98)_ = 0.154	0.695
	Genotype	*F*_(1,98)_ = 0.057	0.812
	Chamber	*F*_(1,98)_ = 116.145	<0.001^∗^
Social novelty			
Chamber time	Phase × genotype × chamber	*F*_(1,98)_ = 3.249	0.075^∼^
	Genotype × chamber	*F*_(1,98)_ = 0.189	0.664
	Phase × chamber	*F*_(1,98)_ = 0.294	0.589
	Phase × genotype	*F*_(1,98)_ = 1.461	0.230
	Phase	*F*_(1,98)_ = 0.133	0.716
	Genotype	*F*_(1,98)_ = 3.638	0.059^∼^
	Chamber	*F*_(1,98)_ = 0.122	0.728
Sniffing time	Phase × genotype × chamber	*F*_(1,98)_ = 0.350	0.556
	Genotype × chamber	*F*_(1,98)_ = 0.054	0.816
	Phase × chamber	*F*_(1,98)_ = 0.042	0.837
	Phase × genotype	*F*_(1,98)_ = 0.057	0.811
	Phase	*F*_(1,98)_ = 0.417	0.520
	Genotype	*F*_(1,98)_ = 2.688	0.104
	Chamber	*F*_(1,98)_ = 10.576	0.002^∗^

^∗^Statistically significant at ^∗^, *p* ≤ 0.05; ^∼^, 0.05 < *p* ≤ 0.10.

### *Fmr1* KO mice display reduced levels of general anxiety during the active phase

**Figure 2.**
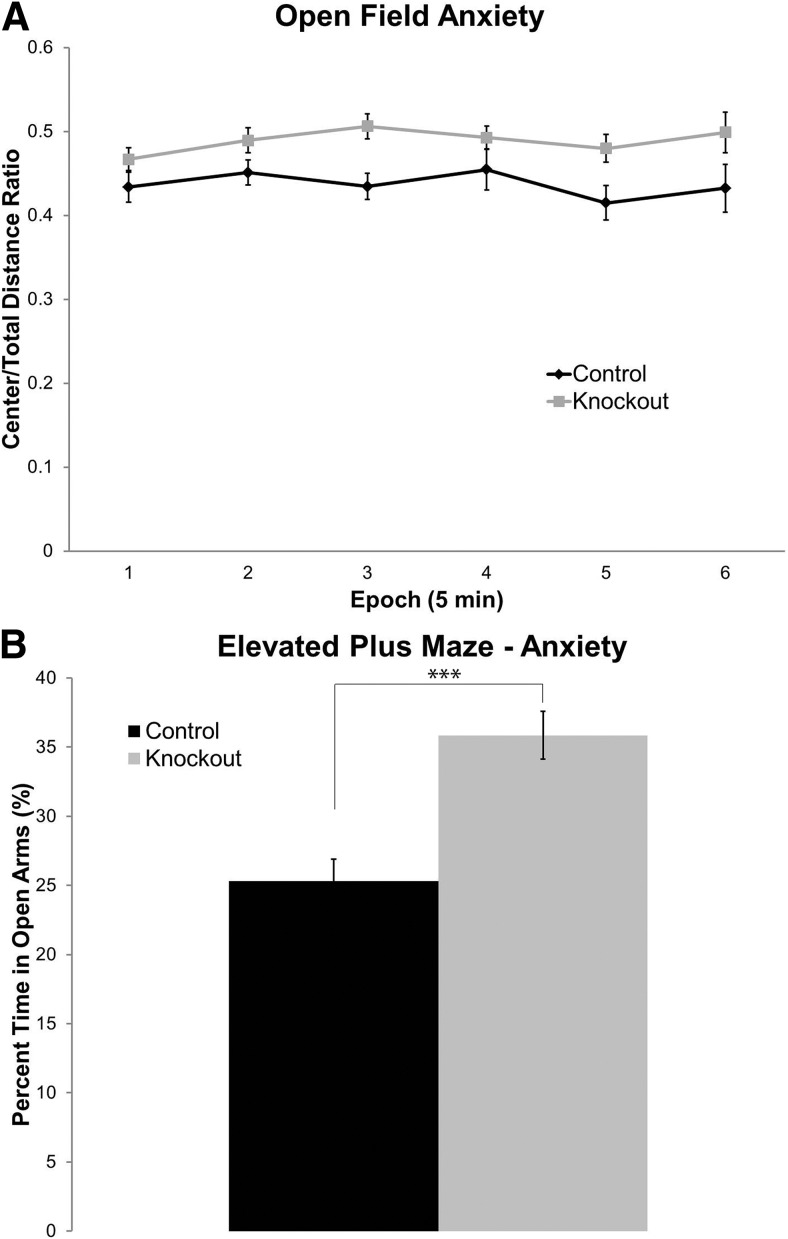
*Fmr1* KO mice display reduced levels of anxiety in the active phase. ***A***, By measuring the ratio of distance traveled in the center to total distance traveled, testing in the open field environment revealed that *Fmr1* KO (n=31) mice had an increased relative distance traveled in the center, indicating reduced anxiety compared with controls (n=25). This is represented by a statistically significant main effect of genotype. ***B***, Testing in the elevated plus maze showed that *Fmr1* KO (n=35) mice spent a significantly greater percentage of time in the open arms compared with controls (n=27) (^∗∗∗^, *p* < 0.001). Each point or bar represents the mean +/− SEM for the number of mice indicated in parentheses.

In the open field test, we determined the ratio of distance traveled in the center to the total distance traveled as an index of anxiety-like behavior. We found a statistically significant main effect of genotype indicating that *Fmr1* KO mice moved more in the center of the field, suggesting reduced general anxiety levels (Table 1, Fig. 2*A*).

As an additional measure of anxiety levels in *Fmr1* KO mice, we determined behavior in the EPM. *Fmr1* KO mice had a significantly increased percentage of time spent in the open arms compared with control mice (*p* < 0.001; Fig. 2*B*), also suggesting reduced general anxiety levels.

### *Fmr1* KO mice have deficits in fear learning during the active phase

**Figure 3.**
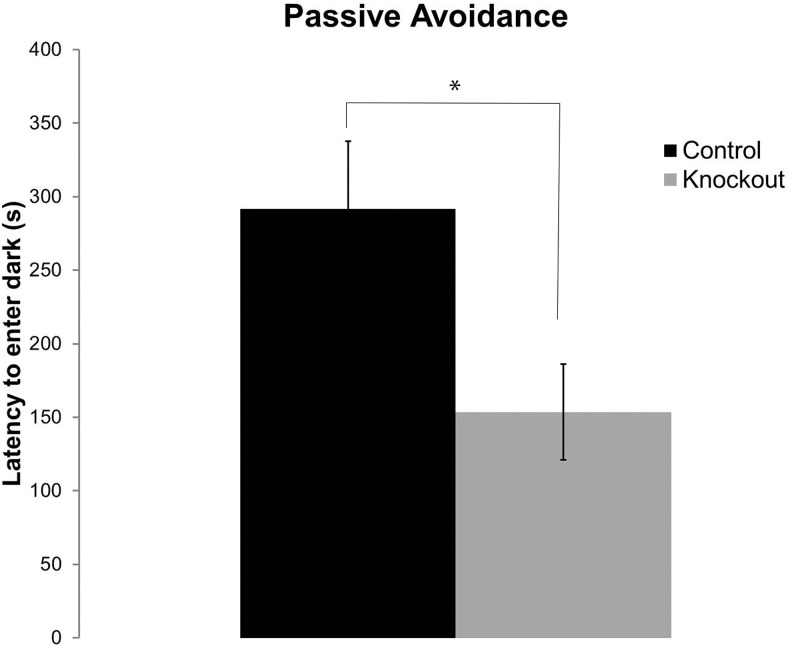
*Fmr1* KO mice display learning and memory impairments in the active phase. Passive avoidance testing showed that *Fmr1* KO (n=34) mice had a significantly reduced latency to enter the dark compared with controls (n=25) (^∗^, *p* = 0.01), suggesting impaired learning and memory. Each bar represents the mean +/− SEM for the number of mice indicated in parentheses.

In the passive avoidance test, we determined the latency to enter the dark chamber during and after training. During the initial training session, the mean latencies were similar for both genotypes; whereas, during the second training session, the mean latency for *Fmr1* KO mice was 40% lower than that of controls. This difference was not statistically significant. The latency to enter the dark compartment during the testing session was significantly lower in the *Fmr1* KO mice compared with controls (*p* = 0.01), suggesting impaired learning and memory (Fig. 3).

### Social behavior is unaffected by circadian phase

The results of initial studies of social behavior conducted in the active phase indicated some differences in *Fmr1* KO phenotype compared with our previous reports (Liu and Smith, 2009; Liu et al., 2011; Qin et al., 2015a,b). We used a slightly altered protocol, so we were uncertain as to whether the differences indicated an effect of circadian phase or were due to the altered procedures. Our previous studies were all conducted with the investigator in the room observing the mouse behavior. In the present studies, we recorded mouse behavior by means of a video camera, and the investigator left the room during testing. To understand these effects, we added testing in the inactive phase [in a separate group of animals maintained on a standard light/dark cycle (lights on at 6:00 A.M.)] to determine whether this was a result of the circadian phase or the change in testing procedures. We conducted social behavior testing in either the inactive or active phases.

**Figure 4.**
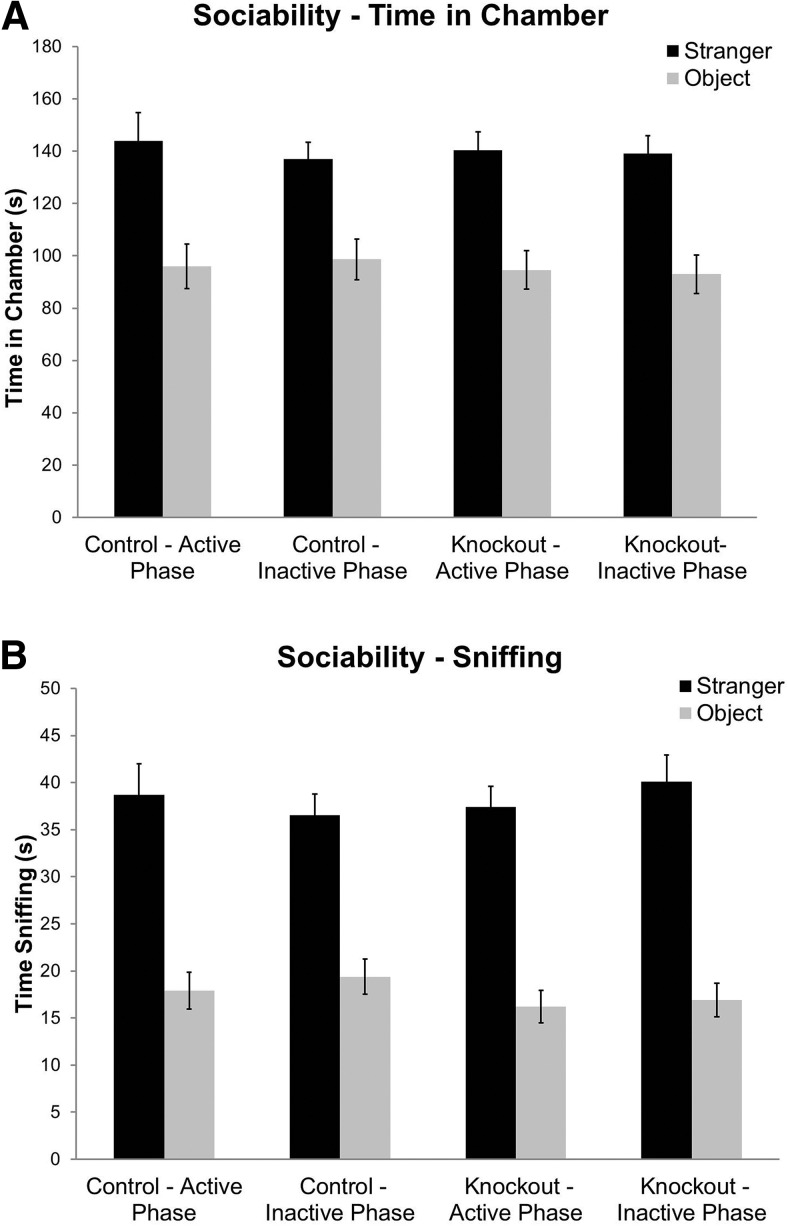
Sociability is unaffected by phase and does not differ by genotype. ***A***, Both control (n=27 active phase) (n=22 inactive phase) and *Fmr1* KO (n=33 active phase) (n=21 inactive phase) mice show sociability based on a preference for time spent in the chamber with a stranger mouse compared with an object. This did not differ by genotype or phase. ***B***, Both control and *Fmr1* KO mice show sociability based on the time spent sniffing a stranger mouse compared with an object. This did not differ by genotype or phase. Each bar represents the mean +/− SEM for the number of mice indicated in parentheses.

In the test for sociability, in which we measured times in the chamber with a stranger mouse or in the chamber with a novel object, both *Fmr1* KO and control mice during either the active or inactive phase showed a preference for the stranger mouse (Table 1, Fig. 4*A*). The only statistically significant effect was a main effect of chamber, indicating that regardless of phase or genotype, mice displayed a preference for the stranger mouse compared with the object. We also analyzed time spent sniffing either the stranger mouse or the object. For this measure also, the only statistically significant effect was a main effect of chamber, indicating no differences in sociability due to genotype or phase (Table 1, Fig. 4*B*).

**Figure 5.**
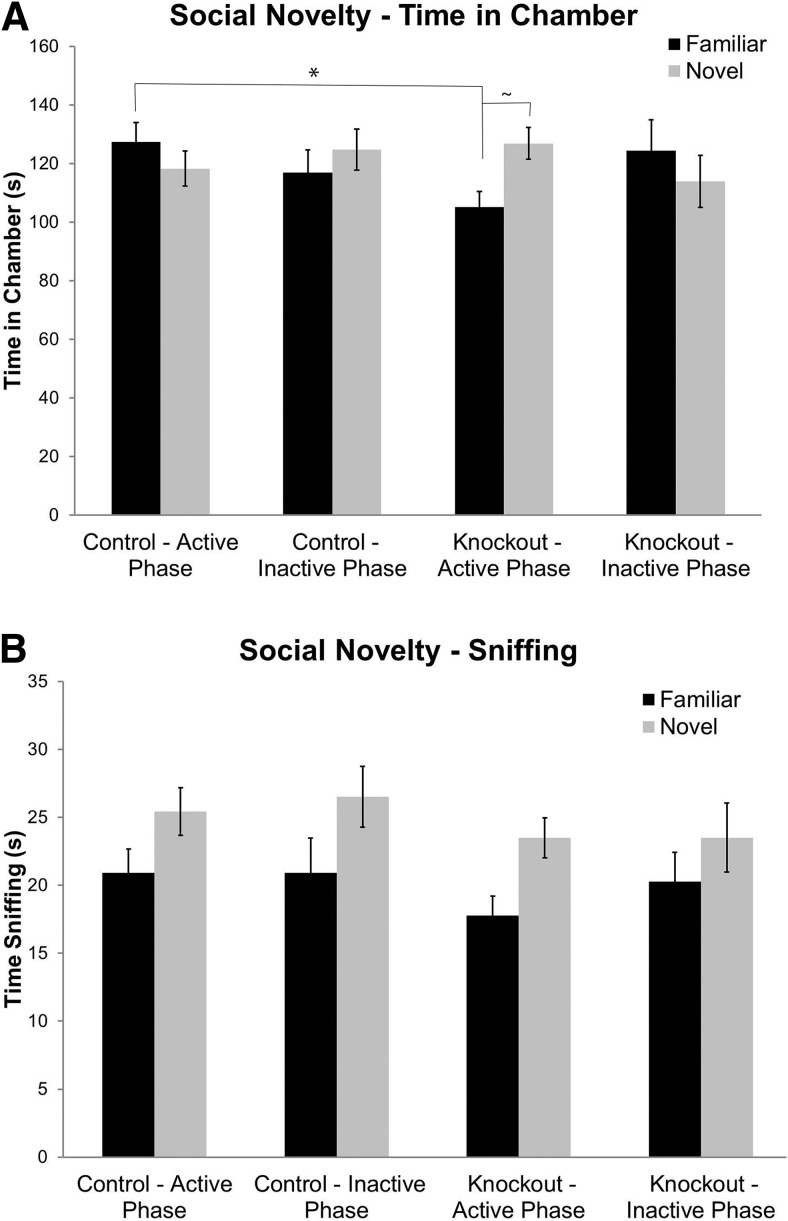
Social novelty may be slightly affected by phase and genotype. ***A***, Time spent in the chamber shows that there was a near significant interaction a phase × genotype × chamber. A *post hoc* pairwise analysis showed a significant difference between the times spent in the chamber with the familiar mouse in the active phase for control (n=27 active phase) (n=22 inactive phase) and *Fmr1* KO (n=33 active phase) (n=21 inactive phase) mice. There was also a near significant preference in the time spent in the chamber with the novel mouse compared with the familiar mouse in the *Fmr1* KO mice during the active phase only. ***B***, Both control and *Fmr1* KO mice showed a preference for social novelty based on the time spent sniffing the novel mouse compared with the familiar mouse. This did not differ by genotype or phase. ^∗^ (p<0.05); ∼ (0.05<p<0.10). Each bar represents the mean +/− SEM for the number of mice indicated in parentheses.

In the social novelty phase of the task, in which the mouse is tested for a preference for either a novel stranger mouse or the now familiar mouse, the phase × genotype × chamber interaction for time spent in the chamber approached statistical significance (*p* = 0.075; Table 1). *Post hoc* pairwise analyses indicate that, in the active phase, *Fmr1* KO mice spent more time in the chamber with the novel mouse than did the familiar mouse (*p* = 0.074), and control mice spent more time in the chamber with the familiar mouse than did *Fmr1* KO mice (*p* = 0.024; Fig. 5*A*). We also analyzed the time spent sniffing the novel and familiar mice. The only statistically significant effect was that of chamber, indicating that, regardless of circadian phase or genotype, there was a preference for sniffing the novel mouse (Table 1, Fig. 5*B*).

## Discussion

We found that in the active phase of the circadian cycle, *Fmr1* KO mice are hyperactive, and demonstrate reduced anxiety and impaired learning and memory. These data are consistent with the behavioral phenotype reported in the inactive phase (Mineur et al., 2002; Qin et al., 2002; Spencer et al., 2005; Ding et al., 2014).

It is a bit surprising that, given the importance of circadian rhythm in physiological functions, it did not have a strong effect on these behaviors. It remains to be seen what, if any, behaviors would be sensitive to circadian phase. It has been suggested that testing during the active circadian phase may increase test sensitivity (Hossain et al., 2004). Perhaps the phenotypes observed in *Fmr1* KO mice are robust enough to be present even in the inactive phase of the mouse. It is possible that conducting a more extensive behavioral battery of tests would reveal more subtle genotype differences observable only during one circadian phase or the other.

One limitation of our study is the need to use illumination during some of the testing, specifically for EPM and passive avoidance tests. There is evidence that the EPM may be influenced by high illumination (Bertoglio and Carobrez, 2002) resulting in decreased exploration of the open arm. However, our EPM data are consistent with the open field data, which were obtained in the dark. Overall results for both EPM and open field tests indicate that the *Fmr1* KO mice displayed decreased anxiety compared with controls, as previously reported in the inactive phase. Another potential limitation of our study is the fact that we shifted the light/dark cycle when mice were 1 month of age. While the 30 d period in the new environment would have allowed enough time for the mice to adjust to the new cycle, the switch was undoubtedly stressful for the mice. Since the behavioral phenotypes were similar in the active phase as previously reported in the inactive phase, it seems unlikely that this stress altered behavior.

For the passive avoidance data, latencies to enter the dark chamber during the first training session did not differ between *Fmr1* KO and control animals, indicating that the baseline exploratory differences were not different. During the second training session, there was a 40% difference in mean latency; *Fmr1* KO mice had a reduced latency to enter the dark. However, these data were highly variable and did not reach statistical significance. After 24 h, during the test phase, latency to enter the dark chamber in *Fmr1* KO mice was statistically significantly lower than that of control mice, indicating impaired learning and memory. Both *Fmr1* KO and control animals vocalized and jumped in response to the foot shock. Studies of *Fmr1* KO mice on a C57BL/6 background indicate normal acute nociceptive responses but reduced nociceptive sensitization (Price et al., 2007). Additionally, acute response to a foot shock did not differ between control and *Fmr1* KO mice (on a C57BL/6J × FVB/NJ mixed background; Nielsen et al., 2009). It is unlikely that performance on this test of learning and memory was a reflection of a genotype difference in pain sensitivity.

We did not detect any differences between genotypes in social behavior, except for a slight increase in preference for social novelty in the active phase for *Fmr1* KO mice. This result suggests that, in the *Fmr1* KO mice, phase may affect social behavior, but the effect was seen only for the measure of time spent in chamber and not for time spent sniffing. In this present study, we did not find any effects of phase or genotype on sniffing time (which is considered to be the more sensitive measure of social behavior). Because the two measures do not show a consistent effect of phase on response to social novelty in *Fmr1* KO mice, we view this as a less robust effect.

Previous studies in which behavior was measured in the inactive phase have reported social behavior deficits in *Fmr1* KO mice, particularly in response to social novelty (Liu and Smith, 2009; Qin et al., 2015a; Sørensen et al., 2015). With our altered protocol, we did not observe genotype differences in social behavior, even in the inactive phase. We interpret this lack of a genotype effect as due to the absence of the experimenter in the testing room, suggesting that even small changes to the behavioral testing procedure (across or within laboratories) can alter the observed phenotype. We conclude that the lack of a social behavior phenotype in *Fmr1* KO mice is not due to circadian phase.

Our results, apart from social behavior deficits, confirm the behavioral phenotype of *Fmr1* KO mice and indicate that they are not a function of circadian phase. These studies validate these mice as reliable models for FXS in which mechanisms of disease pathogenesis and novel therapies may be tested.

## Synthesis

The decision was a result of the Reviewing Editor Cheryl Sisk and the peer reviewers coming together and discussing their recommendations until a consensus was reached. A fact-based synthesis statement explaining their decision and outlining what is needed to prepare a revision is listed below. The following reviewer(s) agreed to reveal their identity: Hongbing Wang

The reviewers and I agree that it is useful to replicate previous behavioral characterizations of the fmr1 KO mouse and to extend these characterizations to different circadian time. However there are some serious concerns about some aspects of the behavioral assessments that you conducted during the dark (active) phase in your experiments. In particular, it seems important to address the potential phase-shifting effects of conducting behavioral tests in a brightly lit room when the animal is in the dark phase, and also how the order of the different tests could affect behavioral outcomes. We appreciate the challenges and complexities involved in characterizing behavior, but think you could do a better job in acknowledging and discussing the limitations of your approach (and these additions would strengthen your paper). Please revise your paper to respond to the specific questions/comments below. In addition, the reviewers noted numerous typographical errors; please carefully proofread your revised manuscript before resubmitting.

1. Behavioral, but also physical and physiological phenotypes are recapitulated in this mouse model. This should be noted in the abstract.

2. Please clarify how the animals for this study were generated. "...generated through heterozygous female and control male breeding pairs maintained in-house" is unclear. KO het * WT (would not give KO...)? Het * Het vs WT*WT (would not be appropriate due to maternal effects and potential genetic drift)? Please specify. How many backcrosses onto B6? What is the original line?

3. Why shift the phase at one month of age? How to control that this late-life shift would not be responsible for behavioral alteration?

4. Some of the tests are conducted in bright rooms. Why this choice? Wouldn't the authors assume this a major perturbation? Of note, mice do not need the room to be bright to distinguish between open and closed arms... and the EPM relies essentially on tactile cues detected by the vibrissae.

5. Why test anxiety last when it is known to be very sensitive (particularly in the EPM), as emphasized by all published recommendations about behavioral testing?

6. The description of the recorded variables is unclear. Many of the variables analyzed are not the ones that have been validated for these paradigms. For instance, in the EPM, the validated variable is the % time spent in the open arms of the EPM [open-arm time/(open-arm time + close-arm time)] not the time spent in open arms.

7. For passive avoidance, what is the interval between habituation, training, and testing?

8. Is there any difference in the number of entrance to the open and closed arm between WT and KO mice?

9. Is there any difference in latency to enter the dark during the passive avoidance training session between WT and KO mice?

10. Is there any difference in sensitivity to the mild shock between WT and KO mice?

## References

[B1] Andrade MM, Tomé MF, Santiago ES, Lúcia-Santos A, de Andrade TG (2003) Longitudinal study of daily variation of rats' behavior in the elevated plus-maze. Physiol Behav 78:125-133. 12536019 10.1016/s0031-9384(02)00941-1

[B2] Bailey DB Jr, Mesibov GB, Hatton DD, Clark RD, Roberts JE, Mayhew L (1998) Autistic behavior in young boys with fragile X syndrome. J Autism Dev Disord 28:499-508. 9932236 10.1023/a:1026048027397

[B3] Bass J (2012) Circadian topology of metabolism. Nature 491:348-356. 10.1038/nature11704 23151577

[B4] Bertoglio LJ, Carobrez AP (2002) Behavioral profile of rats submitted to session 1-session 2 in the elevated plus-maze during diurnal/nocturnal phases and under different illumination conditions. Behav Brain Res 132:135-143. 11997144 10.1016/s0166-4328(01)00396-5

[B5] Chung S, Son GH, Kim K (2011) Circadian rhythm of adrenal glucocorticoid: its regulation and clinical implications. Biochim Biophys Acta 1812:581-591. 10.1016/j.bbadis.2011.02.003 21320597

[B6] Ding Q, Sethna F, Wang H (2014) Behavioral analysis of male and female Fmr1 knockout mice on C57BL/6 background. Behav Brain Res 271:72-78. 10.1016/j.bbr.2014.05.046 24886775 PMC4104211

[B7] Dockendorff TC, Su HS, McBride SM, Yang Z, Choi CH, Siwicki KK, Sehgal A, Jongens TA (2002) Drosophila lacking dfmr1 activity show defects in circadian output and fail to maintain courtship interest. Neuron 34:973-984. 12086644 10.1016/s0896-6273(02)00724-9

[B8] Griebel G, Moreau JL, Jenck F, Martin JR, Misslin R (1993) Some critical determinants of the behaviour of rats in the elevated plus-maze. Behav Proc 29:37-47. 10.1016/0376-6357(93)90026-N 24897695

[B9] Hagerman R, Hoem G, Hagerman P (2010) Fragile X and autism: intertwined at the molecular level leading to targeted treatments. Mol Autism 1:12. 10.1186/2040-2392-1-12 20858229 PMC2954865

[B10] Hagerman RJ, Jackson AW 3rd, Levitas A, Rimland B, Braden M (1986) An analysis of autism in fifty males with the fragile X syndrome. Am J Med Genet 23:359-374. 3953654 10.1002/ajmg.1320230128

[B11] Hossain SM, Wong BK, Simpson EM (2004) The dark phase improves genetic discrimination for some high throughput mouse behavioral phenotyping. Genes Brain Behav 3:167-177. 10.1111/j.1601-183x.2004.00069.x 15140012

[B12] Jones N, King SM (2001) Influence of circadian phase and test illumination on pre-clinical models of anxiety. Physiol Behav 72:99-106. 11239986 10.1016/s0031-9384(00)00388-7

[B13] Kazdoba TM, Leach PT, Silverman JL, Crawley JN (2014) Modeling fragile X syndrome in the Fmr1 knockout mouse. Intractable Rare Dis Res 3:118-133. 10.5582/irdr.2014.01024 25606362 PMC4298642

[B14] Liu ZH, Smith CB (2009) Dissociation of social and nonsocial anxiety in a mouse model of fragile X syndrome. Neurosci Lett 454:62-66. 10.1016/j.neulet.2009.02.066 19429055 PMC3092374

[B15] Liu ZH, Chuang DM, Smith CB (2011) Lithium ameliorates phenotypic deficits in a mouse model of fragile X syndrome. Int J Neuropsychopharmacol 14:618-630. 10.1017/S1461145710000520 20497624 PMC3102293

[B16] Mineur YS, Sluyter F, de Wit S, Oostra BA, Crusio WE (2002) Behavioral and neuroanatomical characterization of the Fmr1 knockout mouse. Hippocampus 12:39-46. 10.1002/hipo.10005 11918286

[B17] Muhle R, Trentacoste SV, Rapin I (2004) The genetics of autism. Pediatrics 113:e472–e486. 15121991 10.1542/peds.113.5.e472

[B18] Nielsen DM, Evans JJ, Derber WJ, Johnston KA, Laudenslager ML, Crnic LS, Maclean KN (2009) Mouse model of fragile X syndrome: behavioral and hormonal response to stressors. Behavi Neurosci 123:677-686. 10.1037/a0015242 19485574

[B19] Price TJ, Rashid MH, Millecamps M, Sanoja R, Entrena JM, Cervero F (2007) Decreased nociceptive sensitization in mice lacking the fragile X mental retardation protein: role of mGluR1/5 and mTOR. J Neurosci 27:13958-13967. 10.1523/JNEUROSCI.4383-07.2007 18094233 PMC2206543

[B20] Qin M, Kang J, Smith CB (2002) Increased rates of cerebral glucose metabolism in a mouse model of fragile X mental retardation. Proc Natl Acad Sci U S A 99:15758-15763. 10.1073/pnas.242377399 12427968 PMC137789

[B21] Qin M, Huang T, Kader M, Krych L, Xia Z, Burlin T, Zeidler Z, Zhao T, Smith CB (2015a) R-baclofen reverses a social behavior deficit and elevated protein synthesis in a mouse model of fragile X syndrome. Int J Neuropsychopharmacol. Advance online publication. Retrieved April 10, 2016. doi:10.1093/ijnp/pyv034. 10.1093/ijnp/pyv034 PMC4576516 25820841

[B22] Qin M, Zeidler Z, Moulton K, Krych L, Xia Z, Smith CB (2015b) Endocannabinoid-mediated improvement on a test of aversive memory in a mouse model of fragile X syndrome. Behav Brain Res 291:164-171. 10.1016/j.bbr.2015.05.003 25979787 PMC5003021

[B23] Schaefer GB, Mendelsohn NJ (2008) Genetics evaluation for the etiologic diagnosis of autism spectrum disorders. Genet Med 10:4-12. 10.1097/GIM.0b013e31815efdd7 18197051

[B24] Scheiermann C, Kunisaki Y, Frenette PS (2013) Circadian control of the immune system. Nat Rev Immunol 13:190-198. 10.1038/nri3386 23391992 PMC4090048

[B25] Sørensen EM, Bertelsen F, Weikop P, Skovborg MM, Banke T, Drasbek KR, Scheel-Krüger J (2015) Hyperactivity and lack of social discrimination in the adolescent Fmr1 knockout mouse. Behav Pharmacol 26:733-740. 10.1097/FBP.0000000000000152 26110222

[B26] Spencer CM, Alekseyenko O, Serysheva E, Yuva-Paylor LA, Paylor R (2005) Altered anxiety-related and social behaviors in the Fmr1 knockout mouse model of fragile X syndrome. Genes brain Behav 4:420-430. 10.1111/j.1601-183X.2005.00123.x 16176388

[B27] Turner G, Webb T, Wake S, Robinson H (1996) Prevalence of fragile X syndrome. Am J Med Genet 64:196-197. 10.1002/(SICI)1096-8628(19960712)64:1&lt;196::AID-AJMG35&gt;3.0.CO;2-G 8826475

[B28] Valentinuzzi VS, Menna-Barreto L, Xavier GF (2004) Effect of circadian phase on performance of rats in the Morris water maze task. J Biol Rhythms 19:312-324. 10.1177/0748730404265688 15245650

[B29] Verheij C, Bakker CE, de Graaff E, Keulemans J, Willemsen R, Verkerk AJ, Galjaard H, Reuser AJ, Hoogeveen AT, Oostra BA (1993) Characterization and localization of the FMR-1 gene product associated with fragile X syndrome. Nature 363:722-724. 10.1038/363722a0 8515814

[B30] Yang M, Weber MD, Crawley JN (2008) Light phase testing of social behaviors: not a problem. Front Neurosci 2:186-191. 10.3389/neuro.01.029.2008 19225591 PMC2622744

[B31] Zhang J, Fang Z, Jud C, Vansteensel MJ, Kaasik K, Lee CC, Albrecht U, Tamanini F, Meijer JH, Oostra BA, Nelson DL (2008) Fragile X-related proteins regulate mammalian circadian behavioral rhythms. Am J Hum Genet 83:43-52. 10.1016/j.ajhg.2008.06.003 18589395 PMC2443847

